# Amplified spontaneous emission and gain in highly concentrated Rhodamine-doped peptide derivative

**DOI:** 10.1038/s41598-021-96982-5

**Published:** 2021-09-02

**Authors:** Andrey Machnev, Daniel Ofer, Ivan Shishkin, Vitali Kozlov, Carlo Diaferia, Antonella Accardo, Giancarlo Morelli, Boris Apter, Alexandra Inberg, Gil Rosenman, Pavel Ginzburg

**Affiliations:** 1 School of Electrical Engineering, Tel Aviv University, 69978 Tel Aviv, Israel; 2 School of Physics & Astronomy, Tel Aviv University, 69978 Tel Aviv, Israel; 3 ITMO University, St. Petersburg, Russia 197101; 4 Department of Pharmacy, Research Centre On Bioactive Peptides (CIRPeB), University of Naples “Federico II”, Via Mezzocannone 16, 80134 Naples, Italy; 5 Faculty of Engineering, Holon Institute of Technology, 52 Golomb st., POB 305, 5810201 Holon, Israel

**Keywords:** Chemistry, Materials science, Optics and photonics

## Abstract

Bioinspired fluorescence, being widely explored for imaging purposes, faces challenges in delivering bright biocompatible sources. While quite a few techniques have been developed to reach this goal, encapsulation of high-quantum yield fluorescent dyes in natural biological forms suggest achieving superior light-emitting characteristics, approaching amplified spontaneous emission and even lasing. Here we compare gain capabilities of highly concentrated *Rhodamine B* solutions with a newly synthesized biocompatible peptide derivative hybrid polymer/peptide material, RhoB-PEG1300-F6, which contains the fluorescent covalently bound dye. While concentration quenching effects limit the maximal achievable gain of dissolved *Rhodamine B*, biocompatible conjugation allows elevating amplification coefficients towards moderately high values. In particular, *Rhodamine B*, anchored to the peptide derivative material, demonstrates gain of 22–23 cm^−1^ for a 10^−2^ M solution, while a pure dye solution possesses 25% smaller values at the same concentration. New biocompatible fluorescent agents pave ways to demonstrate lasing in living organisms and can be further introduced to therapeutic applications, if proper solvents are found.

## Introduction

Bioimaging is a widely used technique, contributing to a broad range of applications, spanning from fundamental cellular studies to applied therapeutics^1^. The ability to visualize individual functional sites within a cell, identify tumors, track conformation processes in real time and many other such abilities make optical tools an inherent part of biomedical research and treatment^2^. Numerous fluorescent dyes have been synthesized over the years such that they cover the entire visible and part of the near-infrared spectrum. While each specific investigation might demand having a certain set of properties, there are several typical requirements common to a majority of bioimaging applications. Those requirements include biocompatibility (or at least a low level of toxicity), high quantum yield and resistance against bleaching and quenching. Biocompatibility issues, inherent to many standard fluorescent dyes, have been addressed with the discovery of green fluorescent protein (GFP)^3^. Since then quite a few GFP derivatives, covering multiple spectral windows, have been developed and have found their use in many bioimaging applications^4^. However, these fluorophores are quite expensive, and thus they may not be the first choice in cases where biocompatibility is not the key characteristic to possess.

Another important property is the capability of dyes to generate a bright emission. This aspect is especially important in cases when optically thick samples, associated with high optical absorbance, are under investigation. Apart from improving quantum yield properties of individual emitters, another possible solution to boosting the emission intensity is to increase the dye concentration. However, a high amount of dye molecules in a small volume can lead to quenching effects. For example, typical concentrations where collective effects start playing a role for Rhodamine dyes are around ~ 10^−2^ mol/L^5–8^, though there is a strong dependence on the solvent used for dye dissolution. In this case, further increasing the concentration does not lead to a boost in the fluorescent signal but rather causes its decrease. The main physical mechanism of the concentration quenching is the aggregation of dye molecules—either due to formation of non-radiative dimers^9^ or of more complex molecular H- and J- aggregates^10^. As a result, an excitation non-radiatively transfers to nearby molecules and dissipates, causing emission quenching.

A possible solution to bypass the concentration quenching is the physical encapsulation of fluorescent molecules into shells, which set a lower cut-off to a proximity parameter. This approach enables elevating the gain and allowed observing an exciton-polaritonic lasing^11^. However, the downside of this biological-type of synthesis is generation of large quantities of fluorescent proteins, which boosts bacterial growth and leads to a consequent material harvesting with relatively low product yield (on scale of hundreds of micrograms)^12^. Thus, a high-throughput process of biocompatible fluorescent dye manufacturing is desirable. The capability to obtain high concentration of fluorescent dyes, which are not a subject to contraction quenching, is a cornerstone for development of new types of dye lasers and even theranostic devices^13^.

Here we investigate a new material, which is a Rhodamine B dye covalently bound to a PEGylated homopeptide of hexaphenylalanine, RhoB-PEG1300-F6. We study the optical properties of this new compound and compare it with a pure Rhodamine B under the same solvent conditions. We show that the Rhodamine conjugation allows preventing concentration quenching effect and provides moderately higher values of gain within an optically pumped solution. It is worth noting, however, that threshold concentrations strongly depend on an embedding environment (typically a solvent) and on the experimental technique which has been used. The latter is linked to a combination of several contributing factors, which complicate the analysis. Consequently, comparative studies of samples, which undergo the same fabrication and characterization path are required—this is what will be done here.

The manuscript is organized as follows—fabrication and basic chemical properties of the new material are discussed first and then followed by spectroscopic characterizations. The latter includes absorption measurements and gain coefficients extraction, which is done by analyzing amplified spontaneous emission dynamics.

## Results and discussion

### Material characterization

**Figure 1:**
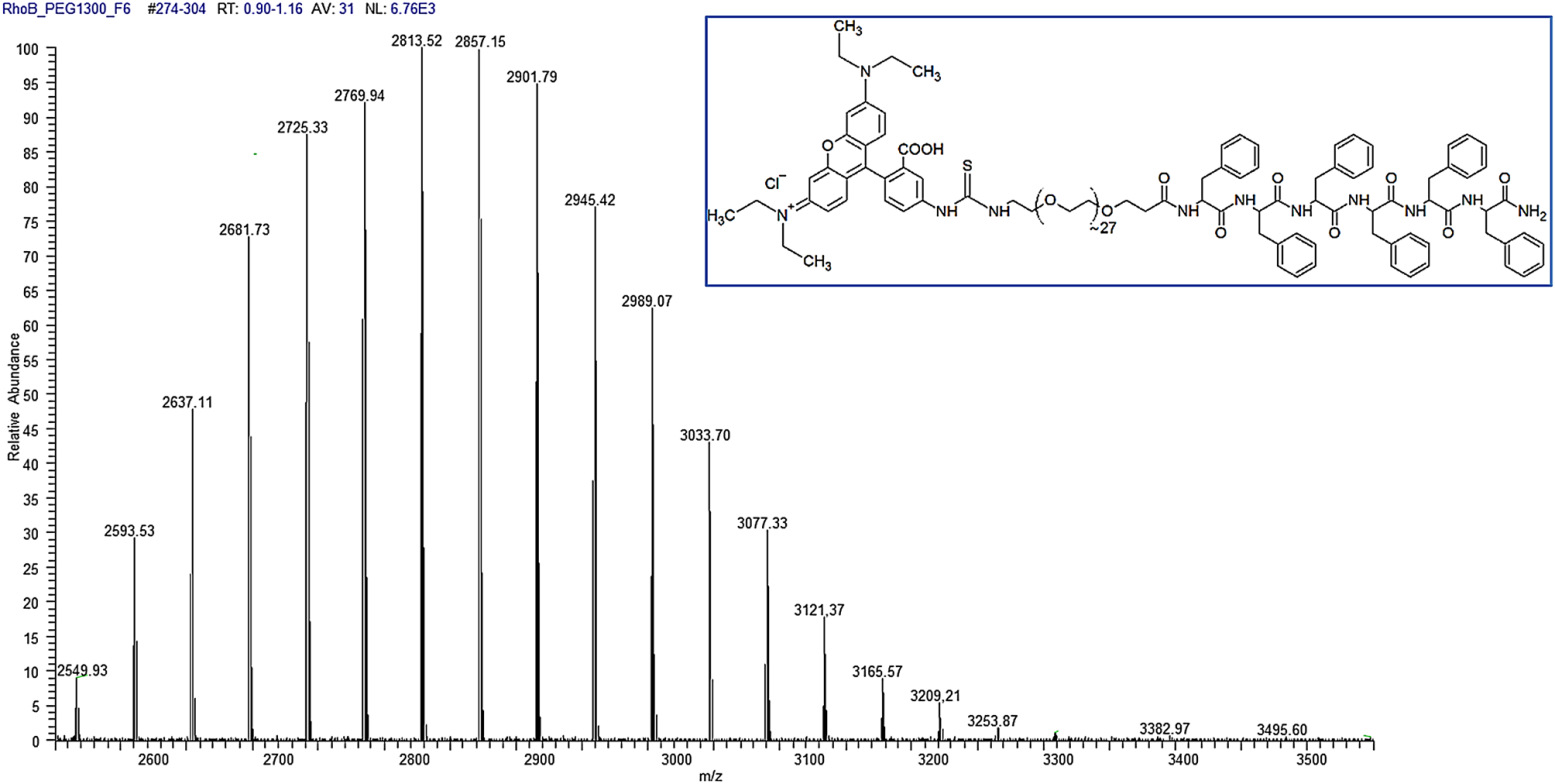
ESI mass spectrum of the Rho-PRG1300-F6 peptide derivative. Inset—schematic representation of Rho-PRG1300-F6 molecule.

Peptide derivative Rho-PEG1300-F6, of which the molecular structure appears in the inset to Fig. 1, was synthetized in a solid phase according to the Fmoc/OtBu strategy^14^, purified by RP-HPLC and characterized by electrospray ionization (ESI) mass spectrometry (Fig. 1). The peptide contains six phenylalanine residues, a polyethylene glycol (PEG) spacer (with an average molecular weight of ~ 1300 Da) and the Rhodamine B fluorescent dye. Due to the aromatic framework (F6), Rho-PEG1300-F6 is poorly soluble in water, whereas it is soluble in 1,1,1,3,3,3-hexafluoro-2-propanol (HFIP) up to 30 mg/mL (~ 1 × 10^−2^ M). This is a well-known disaggregating solvent used to solubilize aromatic peptides with a high propensity to self-assemble into β-sheet structures.

In order to get information on the aggregation state of the peptide derivative in HFIP we performed dynamic light scattering (DLS), circular dichroism (CD) and Fourier Transform Infrared (FTIR) measurements. Analogously, we also characterized the peptide in HFIP/H_2_O (20/80, v/v) mixture, in which peptide arrangement into β-sheet macrostructures is expected. This consideration is based on different studies previously performed on hexaphenylalanine analogues^15–17^. These two solvent conditions will tremendously change fluorescent properties of the material, as we will show hereafter.

**Figure 2:**
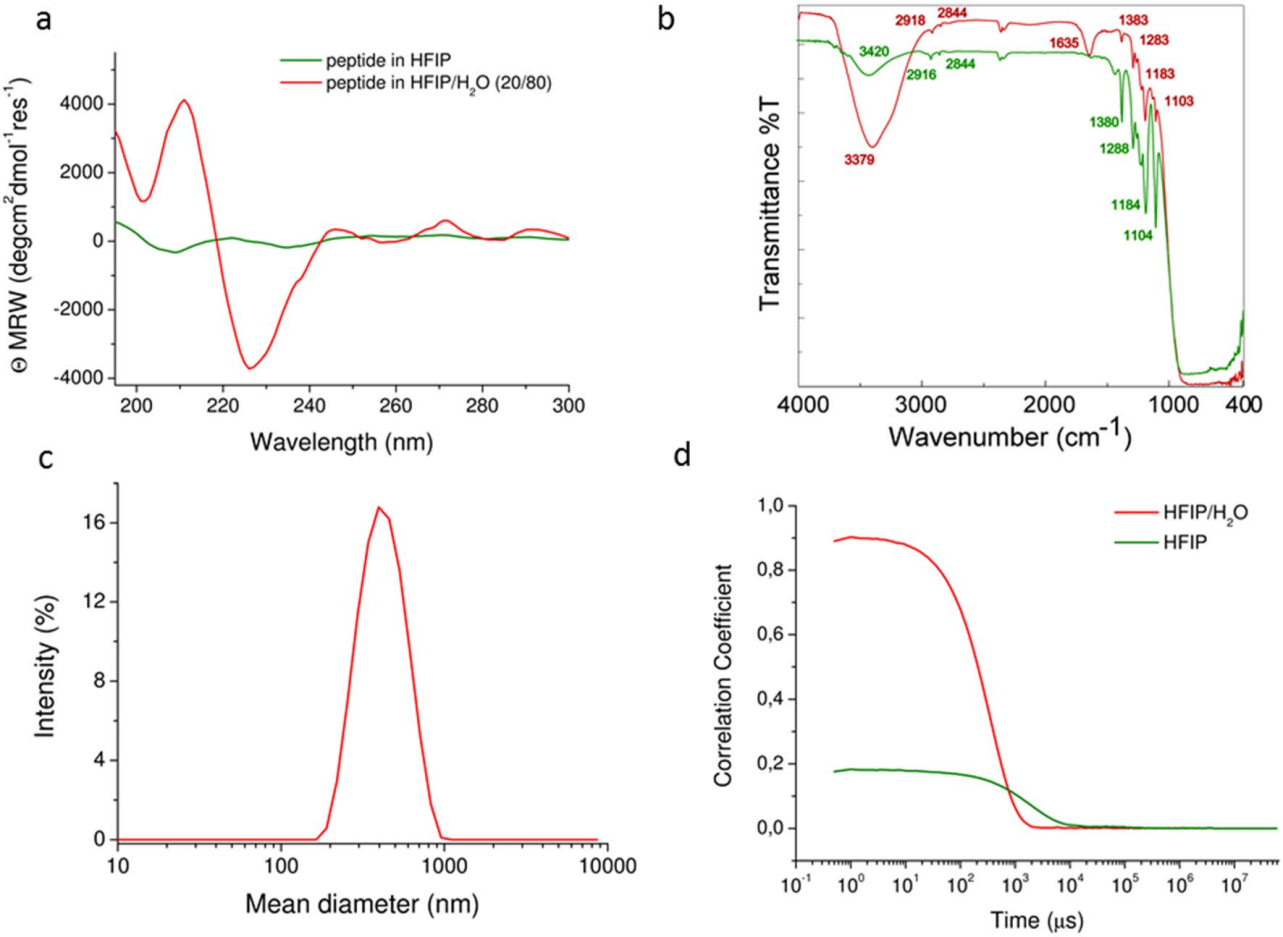
Structural characterization of Rho-PEG1300-F6 in solutions. (**a**) CD spectra of Rho-PEG1300-F6 in HFIP at a concentration of 5 × 10^−3^ M and in HFIP/H_2_O (20/80) at a concentration of 1 × 10^−3^ M. (**b**) FTIR spectra of the peptide in HFIP (green line) and in HFIP/H_2_O (red line) at a concentration of 5 × 10^−3^ M and 1 × 10^-3^ M. (**c**) DLS profile of Rho-PEG1300-F6 in HFIP/H_2_O at a concentration of 1 × 10^−3^ M. (**d**) Correlation functions of the peptide in HFIP and in HFIP/ H_2_O (20/80).

CD spectroscopy is typically employed for monitoring conformational changes of peptide-based nanostructures and mainly for revealing secondary structural features, such as β-sheet structures, accompanying the formation of amyloid fibers (e.g.^18,19^). CD spectrum of Rho-PEG1300-F6 in HFIP/H_2_O clearly shows a maximum around 210 nm, attributable to aromatic side-chains stacking, and a minimum around 227 nm, associated with a β-structure (Fig. 2a). On the contrary, weak CD signals are detectable for peptide derivative in HFIP, thus confirming the lower propensity to the peptide aggregation in this solvent. This result was further confirmed by comparing FTIR spectra of Rho-PEG1300-F6 in HFIP (green line) and in HFIP/H_2_O (red line) at a concentration of 5 × 10^−3^ M and 1 × 10^−3^ M in Fig. 2b. Both the spectra show superimposable peaks attributable to the peptide in the finger print region (between 1400 and 400 cm^−1^), whereas two broad bands, due to the peptide in its aggregated form, are clearly detectable in the amide A and in the amide I regions (at 3379 cm^−1^ and 1635 cm^−1^, respectively) for the sample in HFIP/H_2_O.

DLS also provides quite remarkable results, showing that water dilution of HFIP allows for a macrostructure formation (Fig. 2c). On the other hand, aggressive undiluted HFIP solvent prevents any aggregation and does not allow β-sheet formation. This effect is clearly visible from a comparison of the correlation function of the peptide in the two solvents (Fig. 2d).

In the following sections we will compare pure Rhodamine and Rho-PEG1300-F6, dissolved in HFIP. Similar investigations were made for HFIP/H_2_O solvent, but they did not indicate any optical properties which are worth investigating in the context of this report—the gain characteristics of Rhodamine are completely diminished by β-sheet structures. This secondary structure demonstrates a broadband absorbance over the entire visible spectrum and, as a result, quenches the emission from an encapsulated dye.

### Fluorescence measurements

#### Absorbance

Absorbance spectra of dyes typically indicate the preferable spectral range where the optical pump is most efficient. Absorption measurements were performed on solutions of Rhodamine B and Rho-peptide (short name for Rho-PEG1300-F6 from here on), dissolved in HFIP or in HFIP/H_2_O (20/80). As was previously mentioned, the solvent was chosen to dissolve the new material efficiently and at the same time, prevent emission properties degradation. As we will show afterwards, HFIP does not diminish fluorescent properties, which can be significantly affected by standard alcohol solvents. As an example, methanol solutions of Rho-peptide exhibit extremely poor fluorescent properties, as we had verified.

**Figure 3:**
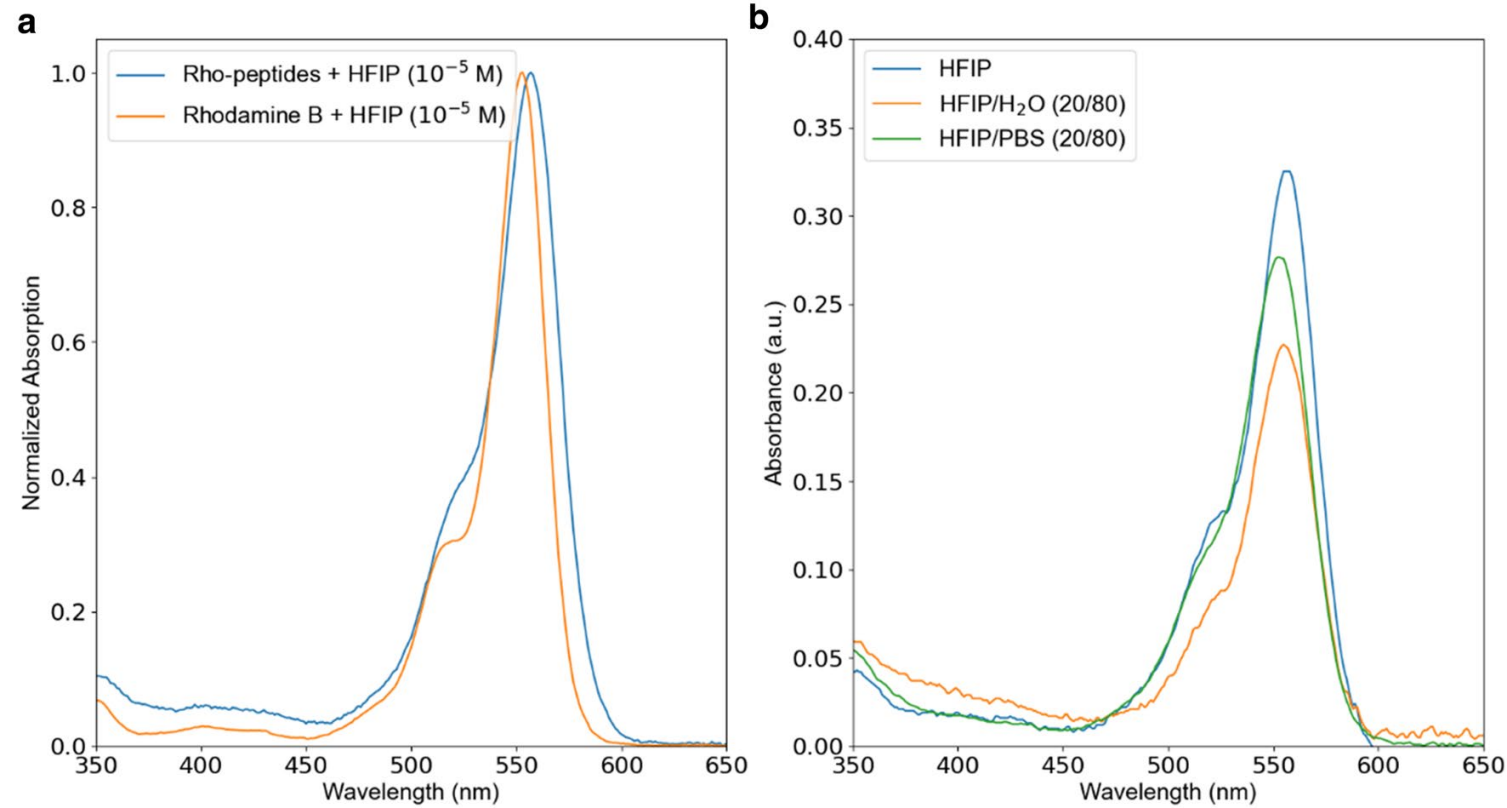
(**a**) Absorbance spectra—Rhodamine B (blue line) and Rho-peptide (orange line). The dyes are dissolved in HFIP with a molar concentration of 10^−5^ M. (**b**) Absorbance spectra of Rho-PEG1300-F6 in HFIP, HFIP/H_2_O (20/80) and in HFIP/PBS (20/80) at pH 7.4. Spectra are recorded between 350 and 650 nm.

Molar concentration of the fluorescent dye in the absorbance experiments was kept at 10^−5^ M. It is also worth noting that the absorption spectra depend on concentration, since molecular dyes tend to aggregate. Several models have been developed to analyze this behavior, e.g.^7^. Here we use moderately low concentration to ensure a minor impact of the probable aggregation. Figure 3a summarizes the results, obtained with a commercial spectrophotometer (macy UV-1200). The maximum absorbance peak of Rho-peptide is red-shifted with respect to the spectral maximum of the pure dye. This phenomenon is most likely associated with the strong polar nature of the solvent, which acts differently on shielded and unshielded dye molecules. In the subsequent studies the samples will be pumped with 532 nm nanosecond laser, which excites the solutions quite efficiently. In order to evaluate the effect of the solvent on the absorbance wavelength another measurement has been done. Figure 3b demonstrates a comparison between the UV–Vis spectra of the Rho-peptide derivative in HFIP, in HFIP/H_2_O (20/80) and in HFIP/PBS (20/80, PBS at a concentration of 0.1 M and pH = 7.4). Absorbance plot of Rho-peptide reveals similar features for all considered solvents—peak around 257 nm occurs owing to absorption in phenylalanine residues while peak at 556 nm is due to the Rho dye. The peak associated with rhodamine is asymmetric with additional local maximum at 525 nm, which appears slightly blue-shifted for the sample in water (λ = 520 nm). Analogous absorbance behavior was observed for the sample in PBS, thus indicating an insignificant impact of the pH variation.

### Variable stripe length method and data fit

**Figure 4:**
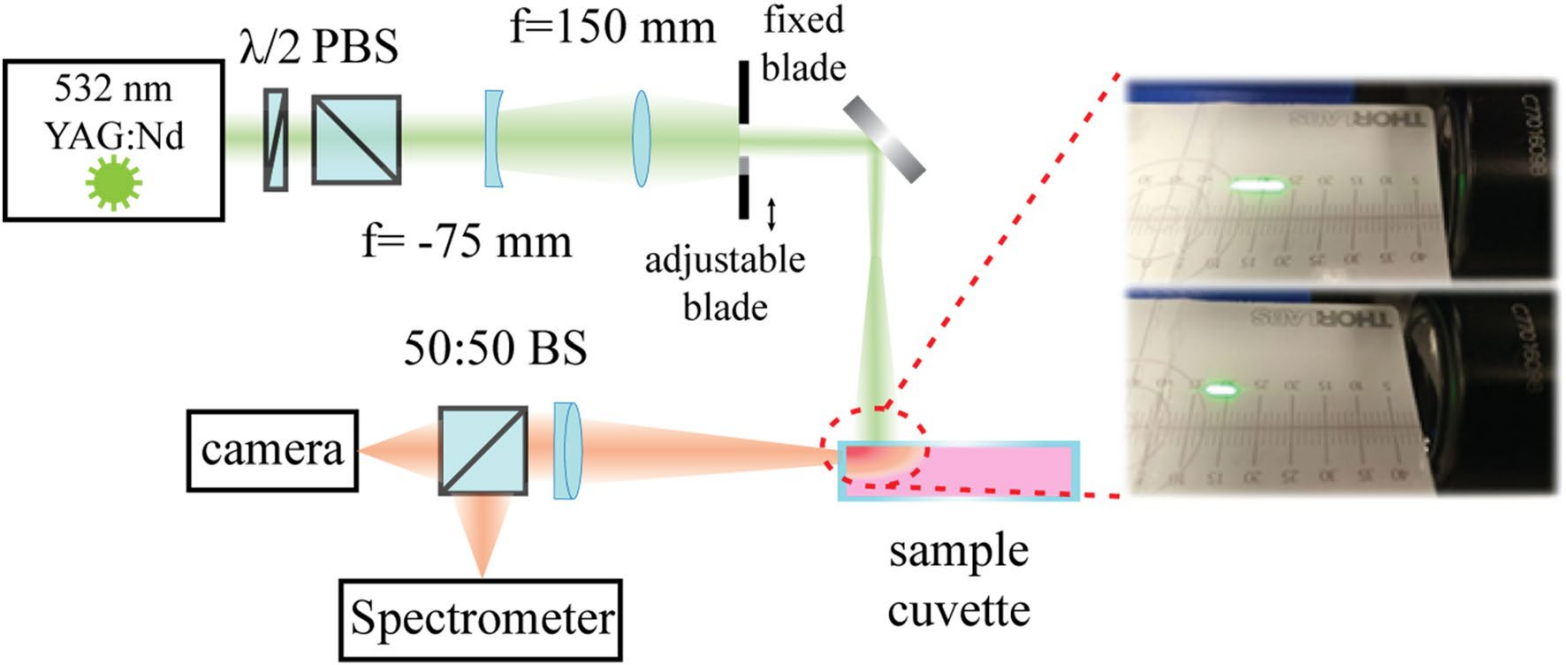
Schematics of the experimental setup. Right insets—photographs of slit illuminations with variable length.

Amplified spontaneous emission (ASE) is a direct indication of gain produced by a pumped medium. Spontaneously emitted photons propagate through an active region and get amplified. The output signal depends on the pump intensity and geometrical factors associated with the collection optics and illumination scheme. Here we used the variable stripe length method (VSLM)^20^ to measure the gain properties of the solution. The essence of the technique is to optically pump a medium through a slit with a controllable variable length. Since ASE signal strongly depends on the propagation length within an active region, the overall gain can be retrieved from straightforward intensity measurements. The fit of the parameters can be based on three different models with an increasing complexity—(i) small signal gain, (ii) homogeneous gain saturation, and (iii) inhomogeneous gain saturation. It is worth noting that the last two models represent different interaction scenarios, where inhomogeneous broadening is most likely to happen in dissolved self-assembled nanomaterials. The set of equations below correspond to the aforementioned scenarios and provide the position-dependent intensity along the strip as follows^21^:

1a $$ \frac{dI\left( z \right)}{{dz}} = g_{0} I\left( z \right) - \alpha I\left( z \right) + C $$

1b $$ \frac{dI\left( z \right)}{{dz}} = \frac{{g_{0} }}{{1 + \frac{I\left( z \right)}{{I_{Sat} }}}}I\left( z \right) - \alpha I\left( z \right) + \frac{C}{{1 + \frac{I\left( z \right)}{{I_{Sat} }}}} $$

1c $$ \frac{dI\left( z \right)}{{dz}} = \frac{{g_{0} }}{{\sqrt {1 + \frac{I\left( z \right)}{{I_{Sat} }}} }}I\left( z \right) - \alpha I\left( z \right) + \frac{C}{{\sqrt {1 + \frac{I\left( z \right)}{{I_{Sat} }}} }} $$

where $I\left(z\right)$ is the light intensity along the strip ($z$ is the length coordinate), ${g}_{0}$ is the small signal gain (including model volume correction factor), $\alpha$ is the linear intensity loss associated with a propagation in the medium, and ${I}_{Sat}$ is the saturation intensity. $C$ is a constant related to the spontaneous lifetime and collection optics as follows: $C={\gamma }_{sp}{N}_{inv}\hslash \omega \frac{\Omega }{4\pi }$. Here ${\gamma }_{sp}$ is the spontaneous emission rate (of a single dye molecule), ${N}_{inv}$ is the density of excited fluorophores (small signal population density), $\hslash \omega$ is an energy of emitted photons, and $\frac{\Omega }{4\pi }$ the normalized stereo angle of the collection optics (refer to Fig. 4 for the setup photograph). While Eqs. 1(a) and 1(b) have a closed form analytical solution, Eq. 1(c) can only be solved numerically. It is also worth noting that Eq. 1(b) approximates 1(c) and 1(a) approximates 1(b) if $I\left(z\right)\ll {I}_{Sat}$. Hence, the comparison between the fit accuracy of real experimental data can allow understanding of how far the gain is from saturation and what is the most-likely broadening mechanism. However, making the comparison requires approaching the saturation intensity, which might be quite challenging because of lasing, which can occur due to an undesirable feedback from elsewhere. The boundary condition for the differential equations is vanishing intensity for the zero-length slit. While the simplest undepleted case (Eq. 1(a)) has a clear exponentially growing form, saturation effects make the functional dependence more complex. Those aspects will be discussed in the context of the experimental data hereafter. It is also worth noting, that numerical fitting of experimental data with the aid of numerical solutions of Eq. 1(c) did not provide any reasonable results owing to a seemingly large flexibility in the unknown parameters.

### Experimental setup

Our experimental setup for ASE measurement is based on pulsed YAG:Nd laser (Litron Nano S) equipped with built-in motorized attenuator and second harmonic generation crystal, which provides excitation energies up to 81 mJ per pulse at 532 nm wavelength. The excitation microstrip was formed using a pair of cylindrical lenses (f = − 75 mm and f = 150 mm). The stripe length is varied by using an adjustable slit, placed in the beam path. Measured solutions were filled to a quartz cuvette. Emitted photoluminescence was collected using an achromatic lens with focus length 50 mm and analyzed using a fiber-coupled spectrometer (Andor Shamrock 193), equipped with CCD camera (iDus 401). The schematics of the setup appear in Fig. 4, while the right insets demonstrate photographs of two different lengths of the excitation stripes.

It is worth noting that VSLM, while being relatively straightforward in experimental realizations, has several disadvantages inherent to its realization. For example, pump diffraction, gain saturation, inhomogeneous collection of the signal and many other effects can affect the results^22^. Furthermore, direct gain measurements close to a saturation point are complicated, since a careful suppression of an optical feedback is needed—otherwise lasing might occur. However, the goal of our investigation is to compare between gain coefficients of two materials, which are kept at exactly the same conditions.

### ASE measurements of Rhodamine and Rho-peptide samples

**Figure 5:**
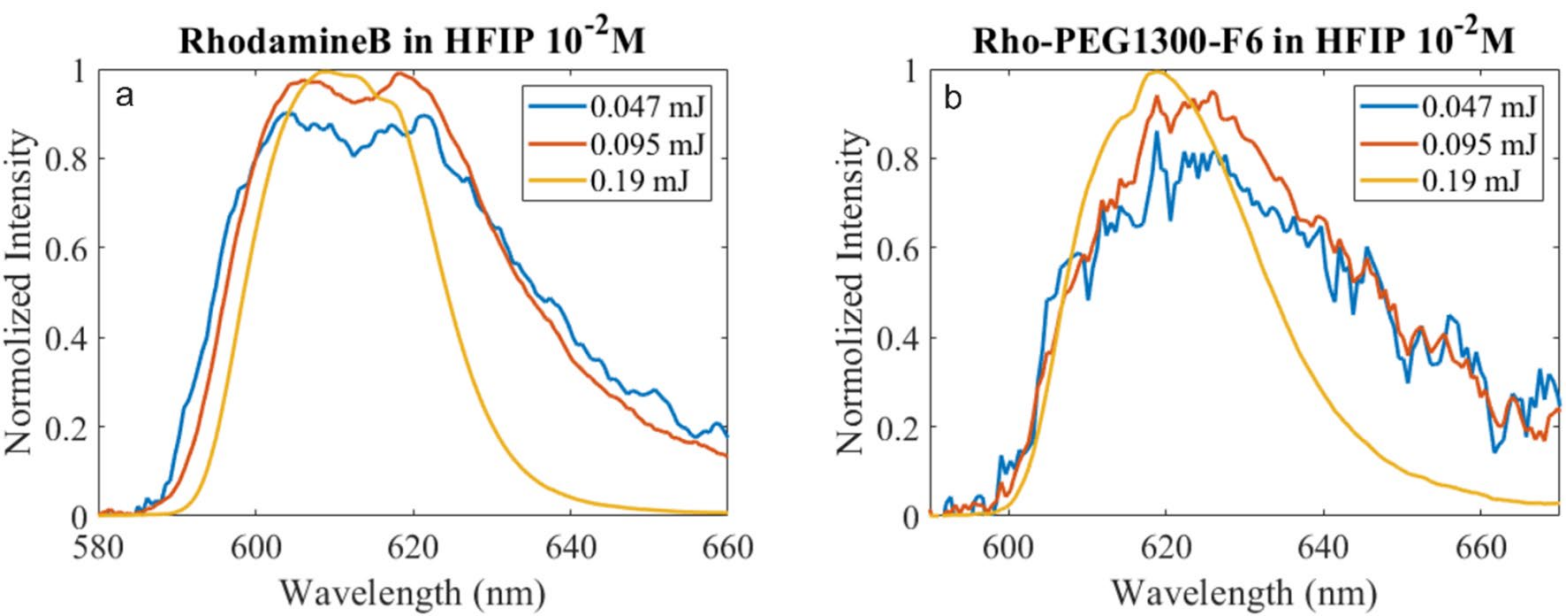
Spectral narrowing of the fluorescence—normalized fluorescent spectra of optically pumped samples. (**a**) Rhodamine B and (**b**) Rho-PEG1300-F6 at 10^−2^ M concentration. Energies per pulse of the pump are indicated in the legends.

In this section we will compare four different samples—HFIP solutions of Rhodamine B and Rho-PEG1300-F6 at two different concentrations—10^−2^ and 10^−3^ M. The first evidence of the ASE and gain is the spectral narrowing of the fluorescent signal with increasing the pumping intensity. For this measurement we kept output laser power constant while varying the slit length—600 µm, 1200 µm and 2400 µm. The corresponding energies per pulse are 0.047 mJ, 0.095 mJ and 0.190 mJ. Figure 5 demonstrates the results—the spectral width is similar for lower pump energies (red and blue curves) and starts decreasing after a certain threshold, where gain regime is reached (red curve). Maximum of the fluorescence occurred at two slightly different wavelengths for rhodamine and Rho-PEG1300-F6 material, 610 nm and 620 nm correspondingly.

**Figure 6:**
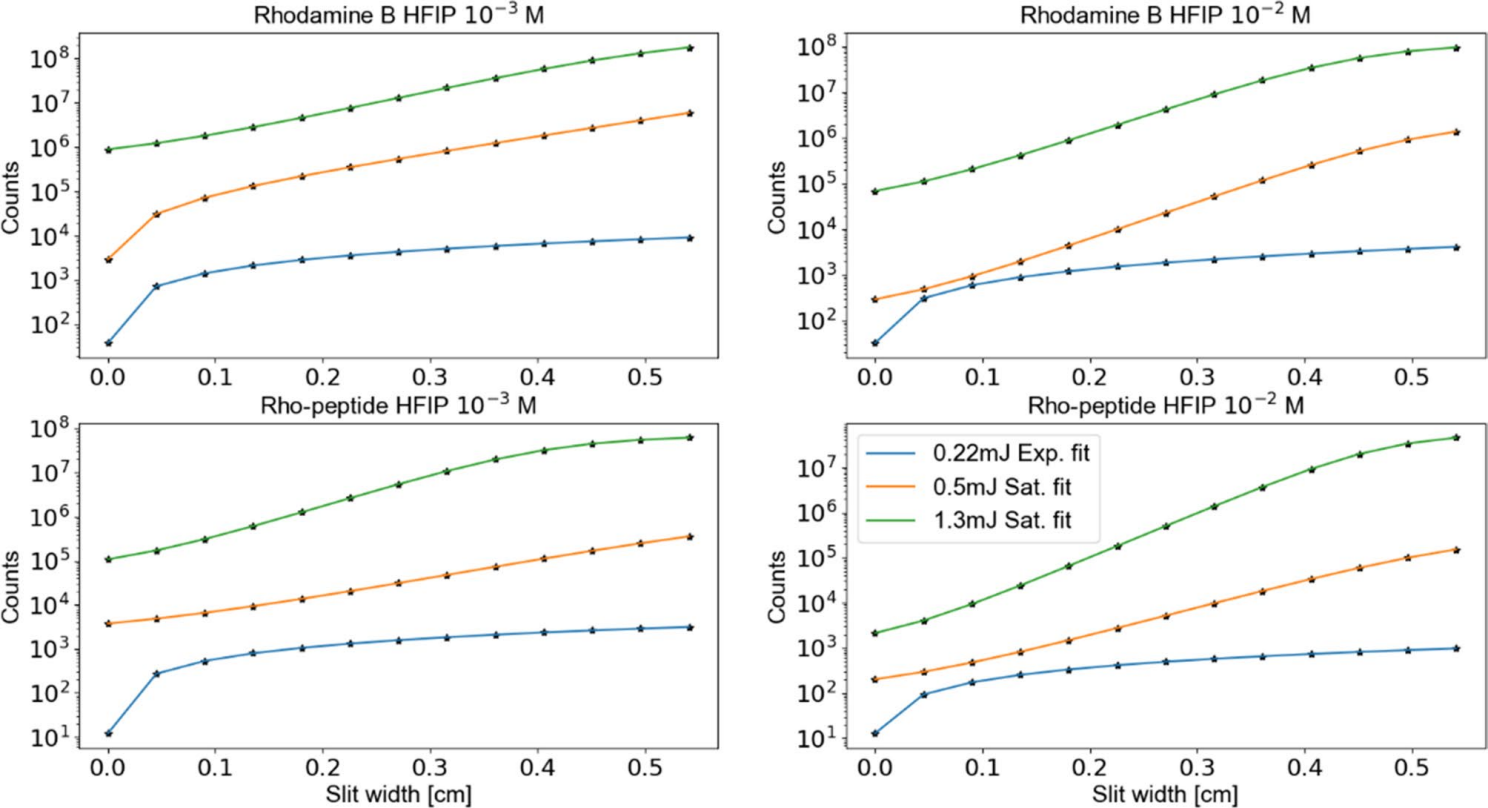
Fluorescence intensity as a function of the slit width. Three pump laser intensities were tested and appear in legends. Numerical data (black dots) is fit with exponential and saturation models – Eqs. 1(a) and (b), respectively. Material, molar concentrations and other information appears in legends and titles.

However, in order to provide a quantitative measure of gain coefficients, fluorescent intensity as a function of the slit width should be analyzed. Figure 6 summarizes the results—those were obtained for HFIP solutions with 10^−2^ and 10^−3^ molar concentrations. Since the gain in the material strongly depends on the pump intensity, three different laser powers were chosen—0.22, 0.5, and 1.3 mJ per pulse. In the case of the smallest pump intensity the gain coefficient is close to 0 and, hence, the best fit was obtained with a simple exponential model (Eq. 1(a)). For the rest of the cases introducing saturation into the model is desirable. We used analytical solutions of Eq. 1(b) for this purpose. In all of the cases, the fit on the logarithmic scale is quite good. It also worth noting that dissolved dyes typically exhibit inhomogeneously broadened behavior, which might be best fitted with Eq. 1(c). The latter, however, does not have an analytical solution and can only be used within a numerical routine. However, owing to a relatively large flexibility in fitting parameters, the results can be quite scattered. This is probably the reason why this methodology in application to dye solutions was not reported in literature to the best of our knowledge.

Gain values extracted from the fitting models are presented in Table 1. The following observations can be made. First, 0.22 mJ is a pump pulse energy, which brings the system close to a transparency point. Second, the exponential fit provides more reasonable results for the lowest pump intensities, while other two values are better fitted with the saturation law. We will use those fits in further discussions. Under lower concentrations (10^−3^ M) both materials provide similar gain coefficients. However, the gain of pure Rhodamine decreases with increase in excitation laser pulse energy (from 0.5 to 1.3 mJ). In this sense, Rho-PEG1300-F6 behaves more linearly with the increase in pump energy. Increasing the molar concentration leads to faster gain increase of Rho-PEG1300-F6 with respect to pure Rhodamine and provides 25% higher gain coefficients. While these results do not provide strong evidence of concentration quenching, they still can be considered as an indication of concentration quenching in Rhodamine solution. For further investigation, net gain spectra^23^ might be measured and used for laser designs.

**Table 1:** Summary of gain coefficients.

Energy	Gain [cm^−1^]	
	Exponential	Saturation
**Rhodamine B 10**^**−3**^ **M**		
0.22 mJ	0.397	10.175
0.5 mJ	8.492	10.590
1.3 mJ	8.299	12.451
**Rho-peptide B 10**^**−3**^ **M**		
0.22 mJ	~ 0	10.885
0.5 mJ	8.460	10.226
1.3 mJ	6.364	17.342
**Rhodamine B 10**^**−2**^ **M**		
0.22 mJ	0.812	9.358
0.5 mJ	11.796	18.705
1.3 mJ	8.314	17.867
**Rho-peptide B 10**^**−2**^ **M**		
0.22 mJ	~ 0	11.658
0.5 mJ	10.899	14.553
1.3 mJ	10.861	22.789

### Photobleaching

An important property of a fluorescent dye is photobleaching, which can significantly degrade gain properties of a solution. This aspect will be studied next.

**Figure 7:**
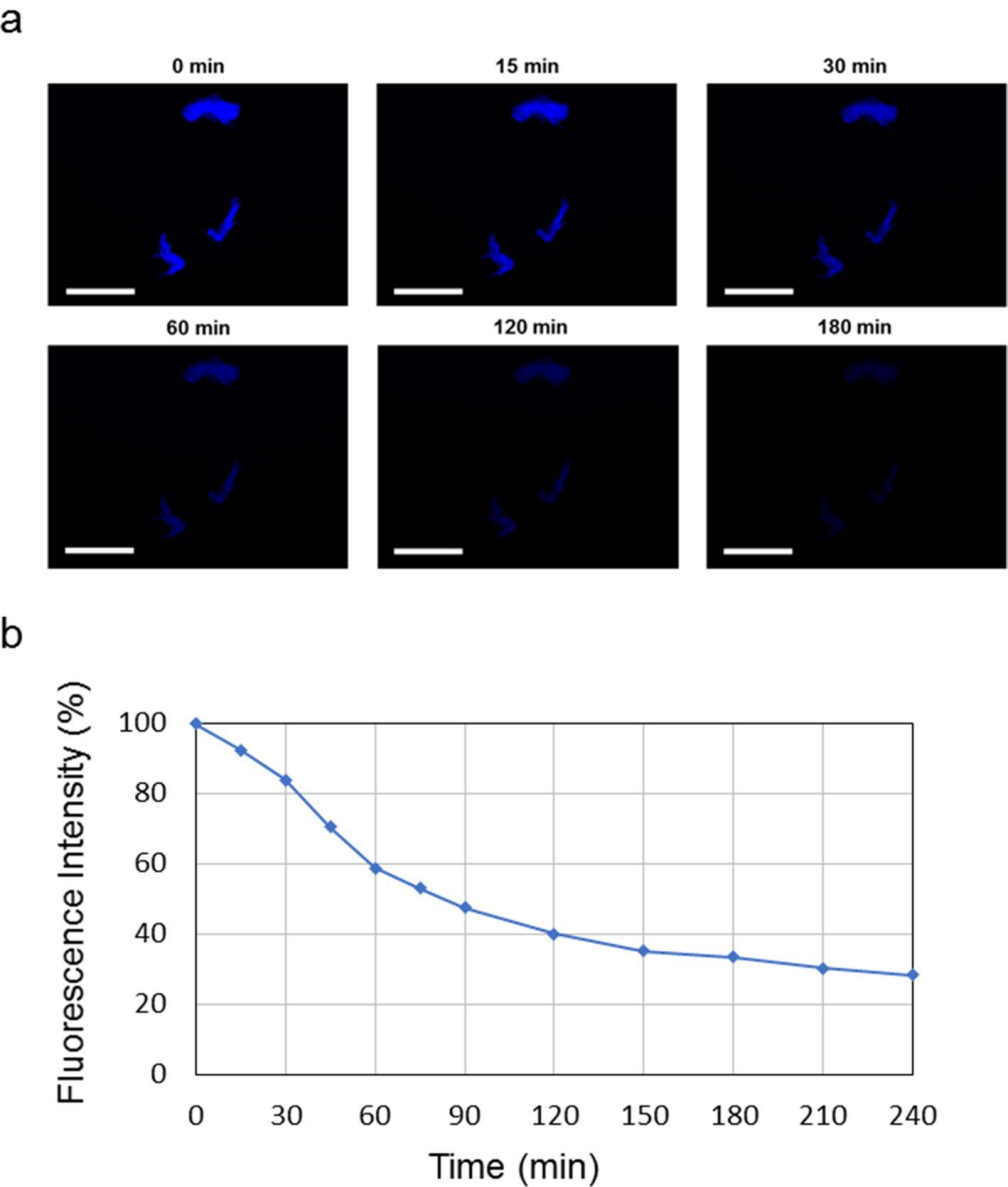
(**a**) Selected fluorescence microscopy images of the peptide solution drop-casted on a glass slide and air-dried at room temperature. Images are obtained by exciting the sample in the spectral region of DAPI and keeping the light on for 240 min. Scale bar is 50 μm. (**b**) Evaluation of the fluorescence intensity percentage decrease due to photoinstability phenomena.

The photostability of Rho-PEG1300-F6 derivative at the solid state was assessed by comparing the fluorescence bleaching rate of the aggregates drop-casted and air dried on glass under continuous excitation for 240 min in the DAPI (4′,6-diamidino-2-phenylindole) spectral region (λexc = 359 nm). Selected immunofluorescence images at different time points indicate that the fluorescence intensity of the dye progressively decreases down to half of the initial value after 90 min (Fig. 7). This bleach rate is much higher than that of some conventional protein dyes^24^ and is in good agreement with the rate previously found for other dye based on aromatic peptides^25^.

## Methodology

### Synthesis of Rho-PEG1300-F6 peptide conjugate

Rho-PEG1300-F6 peptide conjugate was synthesized by according to the standard solid-phase peptide synthesis (SPPS) using 9-fluorenylmethoxycarbonyl (Fmoc) protocols on Rink amide MBHA resin (substitution 0.65 mmol/g). Rhodamine-B-isothiocianate (Rho), Fmoc-PEG1300 (in a polydisperse fashion) and 1,1,1,3,3,3-hexafluoro-2-propanol (HFIP) were purchased by Sigma Aldrich (Milan, Italy).

The elongation of PEG1300-F6 on the resin was achieved as previously reported^14^. After removal of the Fmoc protecting group from the PEG1300, Rho-labeling was performed directly on resin-bound peptides by treating the resin with 2 equivalents of RhoB-isothiocianate in DMF and 4 equivalents of N,N-Diisopropylethylamine (DIPEA). After 24 h, the resin-bound peptide was washed thoroughly with CH_2_Cl_2_ and the peptide was cleaved in acidic condition via trifluoroacetic acid (TFA) using a solution TFA/H_2_O 95/5 v/v and purified by RP-HPLC chromatography. The identity of the Rho-peptide derivative was confirmed by LC–MS analysis assessed using Finnigan Surveyor MSQ single quadrupole electrospray ionization (Finnigan/Thermo Electron Corporation San Jose, CA), column: C18-Phenomenex eluted with an H_2_O/0.1% TFA (A) and CH_3_CN/0.1% TFA (B) from 20 to 80% over 20 min at 200 μL/min flow rate. Retention time, Rt = 18.1 min; MS (ESI +): m/z 1551.28 + 44.05*n*.

### Dynamic light scattering (DLS) measurements

Characterization of Rho-PEG1300-F6 in HFIP (5 × 10^−3^ M) and in HFIP/H_2_O (20/80) (1 × 10^−3^ M) was carried out by DLS measurements using a Zetasizer Nano ZS (Malvern Instruments, Westborough, MA). Instrumental settings for the measurements are a backscatter detector at 173° in automatic modality, room temperature and disposable sizing quartz cuvette as cell. DLS measurements in triplicate were carried out on aqueous samples after centrifugation at room temperature at 13,000 rpm for 5 min.

### Circular dichroism (CD)

Far-UV CD spectra of Rho-PEG1300-F6 in HFIP (5 × 10^−3^ M) and in HFIP/H_2_O (20/80) (1 × 10^−3^ M) were collected on a Jasco J-810 spectropolarimeter equipped with a NesLab RTE111 thermal controller unit at 25 °C using a 0.1 mm quartz cell at 25 °C. The spectra were recorded from 300 to 195 nm. Other experimental settings were: scan speed, 10 nm/min; sensitivity, 50 mdeg; time constant, 16 s; bandwidth, 1 nm. Each spectrum was obtained by averaging three scans and corrected for the blank contribute. Here Θ represents the mean residue ellipticity (MRE), i.e. the ellipticity per mole of peptide divided by the number of amino acid residues in the peptide.

### Fourier transform infrared spectroscopy (FTIR)

Fourier Transform Infrared spectra of the peptide solubilized in HFIP were collected on a Jasco FT/IR 4100 spectrometer (Easton, MD) in an attenuated total reflection (ATR) mode and using a Ge single-crystal at a resolution of 4 cm^−1^. A total of 100 scans for each sample were recorded with a rate of 2 mm s^−1^ against a KBr background. After collection in transmission mode, spectra were converted to emission.

### Photostability study

10 μL of Rho-PEG1300-F6 in HFIP/H_2_O (20/80) at a concentration of 1 × 10^−3^ M were drop-casted on a clean coverslip glass, dried and imaged with fluorescence microscopy. Immunofluorescence images were taken with a Leica DFC320 video-camera (Leica, Milan, Italy) connected to a Leica DMRB microscope equipped with a 10 X and 40 X objectives and the Image J Software (National Institutes of Health, Bethesda, MD) was used for analysis. The photostability was evaluated on a sample left under continuous excitation (spectral region DAPI, (λ_exc_ = 359 nm, λ_em_ = 461 nm) for 240 min and fluorescence images were recorded upon the time at different time points (0, 15, 30, 45, 60, 75, 90, 120, 150, 180 and 240 min). Fluorescence decay was reported as percentage with respect to the amount of the initial fluorescence.

## Outlook and conclusions

Light-matter interaction dynamics in solutions might strongly depend on fluidic interactions. Those typically limit achievable gain parameters in highly-concentrated dye mixtures. Finding pathways to suppress concentration quenching effects can boost efficiencies of dye lasers and make them to be a part of multifunctional theranostic devices.

Here we demonstrate that the conjugation of Rhodamine B to a peptide sequence such as PEG1300-F6 allows elevating the gain quite significantly (25% and more) in respect to pure dye solution. We show that Rhodamine B, dissolved in HFIP, is a subject to quenching at 10^−2^ M concentration. At the same time, the anchorage of the dye on the peptide sequence PEG1300-F6 removes this boundary, paving the way to a new generation of efficient dye lasers. It is worth noting, however, that a proper choice of a solvent should be made if biocompatibility issues might arise.
